# Circulating microRNAs at the Interface of Obesity and Breast Cancer: From Molecular Machinery, Diagnostic Potential, and Therapeutic Perspectives

**DOI:** 10.3390/cancers18183035

**Published:** 2026-09-18

**Authors:** Georgina Victoria-Acosta, Stephanie I. Núñez-Olvera, María Elizbeth Alvarez-Sánchez, Yarely M. Salinas-Vera, Jonathan Puente-Rivera

**Affiliations:** 1 División de Investigación, Hospital Juárez de México, Instituto Politécnico Nacional 5160, Col. Magdalena de las Salinas, Mexico City 07760, Mexico; georgina.acostaa@salud.gob.mx; 2 Departamento de Biología Celular y Fisiología, Instituto de Investigaciones Biomédicas, Universidad Nacional Autónoma de México, Mexico City 04510, Mexico; 3 Laboratorio de Patogénesis Celular Humana y Veterinaria, Posgrado en Ciencias Genómicas, Universidad Autónoma de la Ciudad de México (UACM), San Lorenzo 290, Col. Del Valle, Mexico City 03104, Mexico; maria.alvarez@uacm.edu.mx; 4 Posgrado en Ciencias Genómicas, Universidad Autónoma de la Ciudad de México (UACM), San Lorenzo 290, Col. Del Valle, Mexico City 03104, Mexico; yarely.vera.ms@gmail.com

**Keywords:** breast cancer, obesity, circulating miRNAs, extracellular vesicles, adipose tissue, tumor microenvironment, liquid biopsy, therapy resistance, microRNA therapeutics

## Abstract

Obesity influences breast cancer risk, progression, and therapeutic response through endocrine, inflammatory, metabolic, stromal, and vesicular mechanisms. Circulating and extracellular vesicle (EV)-associated microRNAs are attractive liquid biopsy candidates; however, in obesity-associated breast cancer, they should not be interpreted as tumor-only biomarkers. This narrative review analyzes circulating/EV-miRNAs as mixed host–tumor signals and separates association-based biomarker evidence from functional EV-mediated transfer. We performed a narrative and integrative review of studies on miRNA biogenesis, circulating miRNA transport, EV biology, adipose tissue dysfunction, adipose-tumor communication, biomarker development, therapy response, and methodological reporting standards. The current literature is read along two independent axes: human relevance and source attribution, from non-breast models to obesity-stratified human cohorts, and mechanistic causality, from association only to EV-level function, miRNA-level function, and causality with rescue; clinical validation is treated as a separate qualifier rather than as the top of a single scale. For example, EV-let-7a in overweight/obese breast cancer patients mainly supports an obesity-stratified biomarker candidate. Breast adipose tissue-derived EV cargoes enriched in miR-155-5p, miR-10a-3p, and miR-30a-3p provide stronger functional support for metabolic reprogramming, whereas EV-associated miR-221/222 has mediator-level evidence in endocrine resistance but is not obesity specific. Conclusions: The main contribution of this review is an operational interpretation framework in which circulating miRNAs are evaluated according to probable source, evidence level, and clinical purpose. Future studies should report EV workflow quality, sample type, hemolysis and platelet control, normalization strategy, external validation, tumor subtype, and obesity phenotyping beyond BMI, including waist circumference or waist-to-hip ratio, insulin resistance, diabetes, menopausal status, and inflammatory markers.

## Simple Summary

Obesity can change the environment surrounding breast tumors and may influence how cancer cells grow, spread, and respond to treatment. microRNAs circulate in the blood and can be transported inside extracellular vesicles, which are tiny particles released by cells. These miRNAs may not only come from tumor cells, but also from fat tissue, immune cells, blood cells, and other components of the body. This review explains how circulating and extracellular vesicle-associated miRNAs may connect obesity, inflammation, altered metabolism, and breast cancer progression. It also discusses their potential use as non-invasive biomarkers and as possible therapeutic targets. A central message is that future studies should not interpret these miRNAs as tumor-only signals, but as mixed host–tumor signals that require careful metabolic and technical evaluation.

## 1. Introduction

Breast cancer remains one of the most prevalent malignancies worldwide and continues to represent a major cause of cancer-related morbidity and mortality among women. Global cancer estimates indicate that breast cancer is among the leading cancers in incidence, reflecting both its high population burden and its biological heterogeneity. Recent breast cancer statistics further emphasize that clinical outcomes are shaped by tumor subtype, access to early detection, molecular classification, treatment exposure, and host-related biological factors [1,2].

Obesity has emerged as one of the most important host-related modifiers of breast cancer risk, progression, treatment response, and survivorship. Its biological influence is especially evident after menopause, but it extends beyond risk estimation and includes inflammation, endocrine disruption, insulin resistance, adipokine imbalance, stromal remodeling, and metabolic support of tumor cells [3,4,5]. Epidemiological and translational studies indicate that body mass index (BMI), body composition, central adiposity, postdiagnosis body fatness, and weight change can influence breast cancer outcomes [6,7,8]. Therefore, BMI should be interpreted together with more informative metabolic or anthropometric descriptors, such as waist circumference (WC), waist-to-hip ratio (WHR), insulin resistance, menopausal status, and inflammatory markers, because these variables can influence circulating miRNA profiles independently of tumor burden [9,10].

This metabolic context is essential for interpreting circulating miRNAs because local adipose dysfunction can release EVs and soluble signals capable of reshaping tumor-cell bioenergetics, inflammatory tone, and stromal communication. In this review, these mechanistic examples are reserved for the dedicated adipose EV and therapy-response sections. In this context, circulating microRNAs (miRNAs) have become highly relevant because they connect molecular communication with liquid biopsy. miRNAs are small non-coding RNAs that regulate gene expression at the post-transcriptional level and participate in proliferation, apoptosis, angiogenesis, immune regulation, metabolism, epithelial–mesenchymal transition, invasion, metastasis, and therapy resistance [11,12,13,14,15]. In blood, miRNAs are protected from degradation through association with EVs, Argonaute proteins, or lipoprotein complexes, making them attractive candidates for non-invasive biomarker studies [16,17,18,19].

**Figure 1:**
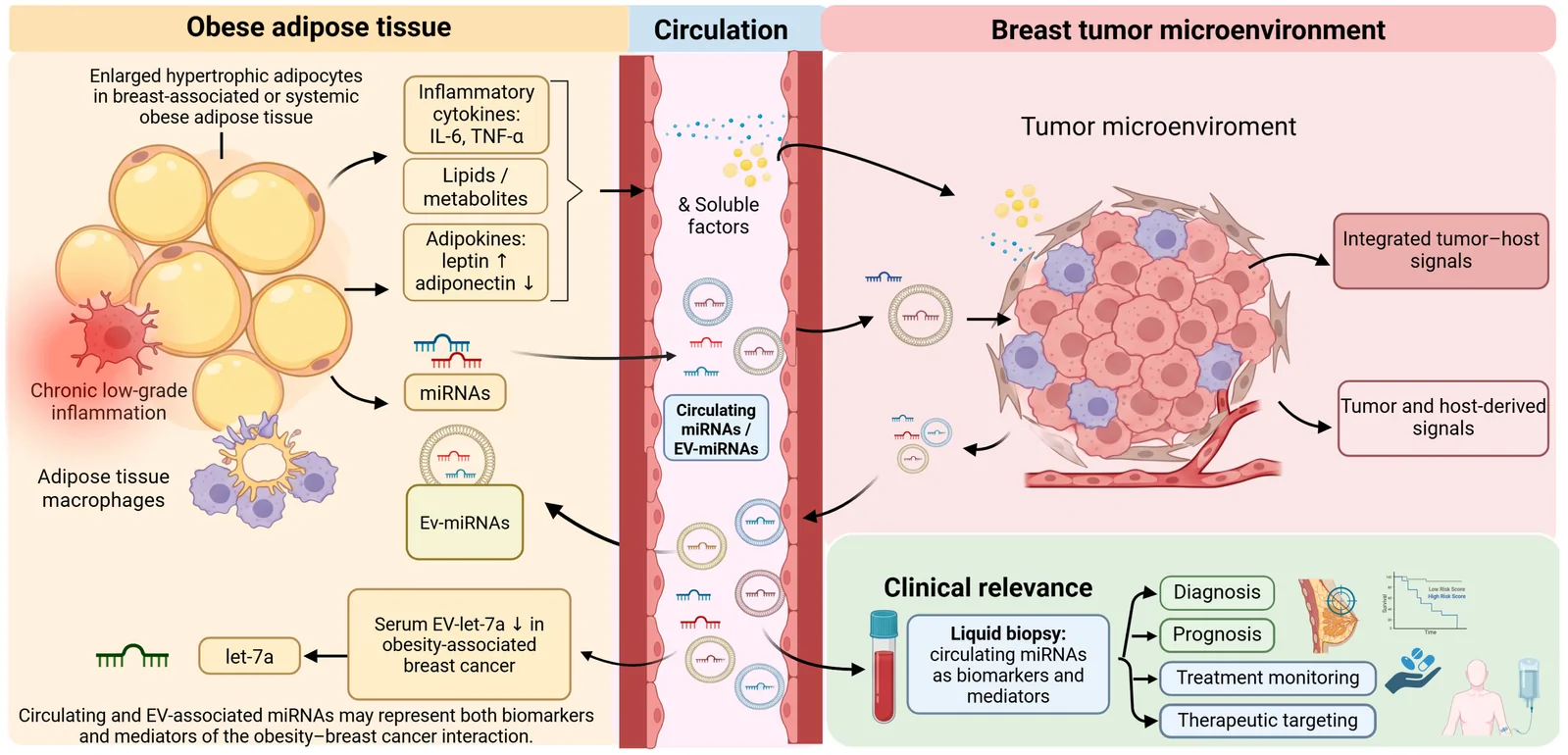
Obesity–breast cancer–circulating miRNA axis. Obese adipose tissue releases adipokines, inflammatory cytokines, metabolites, lipids, and extracellular vesicles carrying miRNAs. These signals interact with breast cancer cells, immune cells, fibroblasts, endothelial cells, and stromal components of the tumor microenvironment, contributing to metabolic adaptation, inflammation, invasion, metastasis, and therapy resistance. Circulating and extracellular vesicle-associated miRNAs may therefore represent both biomarkers and mediators of the obesity–breast cancer interaction. Actionable reading: interpret circulating miRNAs by probable source (tumor, adipose, immune, endothelial, platelet, or mixed) and by evidence tier before making biomarker or mechanistic claims.

The central novelty of this review is to frame circulating miRNAs in obesity-associated breast cancer as mixed-source host–tumor signals rather than tumor-only liquid biopsy markers. We also separate biomarker-type evidence from functional EV-mediated transfer, so that association-only miRNAs are not overinterpreted as causal mediators unless uptake, phenotype induction, and gain- or loss-of-function directed at the miRNA itself are available; interventions acting on the whole vesicle preparation or on a downstream pathway are informative about the EVs but do not identify the miRNA responsible. Finally, we argue that obesity phenotyping is not an optional clinical descriptor but a mandatory confounder control for circulating miRNA studies; BMI should be complemented, when possible, with waist circumference or waist-to-hip ratio, insulin resistance, menopausal status, inflammatory markers, and treatment context. This interpretative axis is summarized in Figure 1. Sepúlveda et al. [20] reviewed EV-miRNA-mediated communication in the breast tumor microenvironment, whereas Cavaleri et al. [21] experimentally investigated EV-mediated crosstalk between adipocyte and breast cancer cell models. Building on these related contributions, this review focuses on circulating miRNAs in obesity-associated breast cancer and integrates metabolic phenotyping with two independent evidence axes: human relevance/source attribution and mechanistic causality. The framework incorporates recent human and translational evidence on obesity-associated EVs in breast cancer, including circulating small EV protein cargo such as ECM1 [22], insulin resistance-stratified plasma EV effects in triple-negative disease [23], and adipose-derived EV cargoes that engage TGF-β/SMAD- and fatty acid-related programs beyond miRNAs [21].

## 2. Obesity and Breast Cancer: Biological and Clinical Context

Obesity is a multifactorial condition characterized by excessive adipose tissue accumulation and profound alterations in endocrine, metabolic, and inflammatory homeostasis. In breast cancer, the association with obesity is not uniform and depends on menopausal status and tumor subtype: excess adiposity is consistently associated with an increased risk of postmenopausal hormone receptor-positive disease, whereas in premenopausal women a higher body mass index has been associated with an unchanged or even lower risk of hormone receptor-positive tumors. Irrespective of menopausal status, obesity is associated with adverse clinicopathological features, poorer prognosis, and reduced therapeutic responsiveness through several interconnected biological mechanisms. One of the most extensively studied pathways involves estrogen biosynthesis. In postmenopausal women, adipose tissue becomes a major site of estrogen production through aromatase-mediated conversion of androgens to estrogens. Obesity-associated adipose inflammation further amplifies this process because inflammatory mediators, including TNF-α, IL-1β, IL-6, and COX-2/PGE2, can induce aromatase expression in adipose tissue, thereby increasing local and systemic estrogen availability [24,25]. Obesity is also frequently accompanied by insulin resistance and compensatory hyperinsulinemia, which may enhance signaling through insulin and IGF-1 pathways. In parallel, obesity alters circulating adipokine profiles, typically increasing leptin levels while reducing adiponectin concentrations. Together with chronic low-grade inflammation and oxidative stress, these endocrine and metabolic disturbances create a systemic environment that may favor tumor initiation and progression, and therapeutic resistance through effects on cell proliferation, survival, angiogenesis, and immune regulation [26,27,28,29,30,31] and these factors converge on PI3K/Akt/mTOR, Ras/Raf/MAPK, NF-κB, HIF-1α [29,30,31]. At the signaling level, obesity-associated circulating factors, including insulin, IGF-1, leptin, and inflammatory cytokines, may converge on PI3K/Akt and MAPK pathways and enhance nongenomic estrogen receptor crosstalk in breast cancer cells, supporting proliferative and progression-related phenotypes in vitro [32]. Consistently, germline polymorphisms in genes of the PI3K-AKT-mTOR pathway have been associated with breast cancer disease-free survival in the context of obesity-related host factors, suggesting a possible interaction between inherited pathway variation, metabolic status, and clinical outcome [33]. More recent integrated miRNA-mRNA analyses in triple-negative breast cancer stratified by obesity further indicate obesity-associated transcriptomic differences in pathways relevant to tumor progression, although these data should be interpreted as associative and not as proof of causality [34].

The adipokine-rich inflammatory milieu may also involve leptin-related JAK2/STAT3 signaling, with SOCS3 acting as a feedback regulator of leptin and cytokine signaling. This axis has been linked to breast cancer cell proliferation, angiogenesis-related programs, and obesity-associated tumor biology [35,36]. In addition, breast adipose tissue-derived extracellular vesicles from women with obesity provide translational evidence that the obese mammary microenvironment can directly alter tumor cell metabolism, mitochondrial activity, and oxidative phosphorylation-related programs [37].

Beyond its systemic effects, obesity profoundly alters the biological properties of mammary adipose tissue. The breast adipose compartment is not merely structural support for the mammary gland but rather a dynamic signaling niche that participates in endocrine, autocrine/paracrine, immune-metabolic, and extracellular vesicle-mediated communication. Adipocytes and stromal vascular cells secrete adipokines, steroid hormones, inflammatory cytokines, complement-related mediators, lipids, metabolites, and extracellular vesicles that contribute to local tissue homeostasis and influence systemic metabolic regulation [38,39,40,41,42]. In obesity, adipocyte hypertrophy, tissue hypoxia, extracellular matrix remodeling, and immune-cell infiltration and metabolic stress collectively shift mammary adipose tissue toward a chronic inflammatory state. One of the most characteristic histological manifestations of this process is the formation of crown-like structures of the breast (CLS-B), defined by macrophages surrounding dead or dying adipocytes. CLS-B are widely recognized as indicators of local adipose tissue inflammation and have been associated with increased aromatase activity, obesity-related breast cancer biology and adverse clinicopathological features [43,44,45,46,47]. This dysfunctional adipose microenvironment may promote tumorigenesis through multiple mechanisms, including sustained inflammatory signaling, enhanced estrogen production, altered adipokine secretion, adipose–tumor crosstalk, and extracellular vesicle-mediated transfer of regulatory molecules, including miRNAs, to tumor and stromal cells [42].

Obesity-associated changes in mammary adipose tissue contribute to extensive remodeling of the breast tumor microenvironment. Cancer-associated adipocytes, adipose-derived stromal/stem cells, macrophages, fibroblasts, endothelial cells, and tumor cells engage in continuous bidirectional communication through adipokines, metabolites, cytokines, lipids, extracellular matrix components, and extracellular vesicles. This reciprocal communication may increase tumor cell plasticity and support several hallmarks of cancer progression. Experimental and translational studies suggest that obesity-associated microenvironmental alterations can promote epithelial–mesenchymal transition, support metastatic dissemination, and modify response to endocrine and cytotoxic therapies [10,48,49,50].

The leptin–JAK2/STAT3–SOCS3 axis described above has also been implicated in obesity-associated breast cancer progression [35,36]. Evidence from preclinical and translational studies supports the concept that obesity reshapes breast cancer biology through coordinated metabolic, endocrine, inflammatory, and microenvironmental mechanisms. In diet-induced obese MTB/TAN mice, obesity is accompanied by increased adiposity, hyperinsulinemia, impaired glucose tolerance, altered adipokine profiles, and dysregulated IGF-1 signaling. These findings suggest that obesity-associated breast cancer recurrence may arise from the combined action of multiple interconnected pathways rather than from a single biological mechanism [51]. Likewise, studies using the PyMT/MMTV model have demonstrated that obesity-induced mammary adipose inflammation can influence mammary tumorigenesis; although the magnitude and direction of these effects may depend on factors such as menopausal status, timing of diet exposure, tumor subtype, and study design [52].

The human functional evidence for adipose EV-mediated metabolic reprogramming of breast cancer cells is presented in Section 5 [37]. These findings are consistent with a growing body of evidence supporting extracellular vesicles as key mediators of adipose-tumor crosstalk within the breast tumor microenvironment [53,54]. Emerging evidence also suggests that obesity may promote DNA damage in mammary epithelial cells before overt malignant transformation occurs, supporting the notion that obesity-associated microenvironmental alterations may contribute to early tumor-promoting events [55].

Although BMI remains widely used for epidemiological stratification, it does not fully capture the biological complexity of obesity. Measures such as WC, WHR, visceral and subcutaneous adipose tissue, imaging-based body composition, insulin resistance, diabetes, menopausal status, adipokine imbalance and systemic inflammation provide a more comprehensive framework for understanding obesity-associated breast cancer biology [9,56,57,58,59,60]. Recent transcriptomic and integrated miRNA-mRNA analyses have identified obesity-associated molecular differences in breast tumors, including pathways involved in metabolism, inflammation, cell signaling, and tumor progression. While these studies remain largely associative, they support the concept that obesity influences gene-regulatory networks relevant to breast cancer development and progression [34]. Collectively, current evidence indicates that obesity is not merely a comorbidity but a biologically heterogeneous condition capable of reshaping many biological processes relevant to breast cancer [56]. Importantly, many of these processes regulate extracellular vesicle biology and miRNA expression, suggesting that obesity may substantially influence circulating miRNA profiles independently of tumor burden [37,54,61]. Therefore, obesity-related variables should be carefully considered in studies evaluating circulating miRNAs as biomarkers in breast cancer to minimize confounding effects and improve biological interpretation.

## 3. Molecular Machinery of Circulating miRNAs and Extracellular Vesicles and Implications in Obesity and Breast Cancer

Most miRNAs are generated through the canonical biogenesis pathway. They are initially transcribed by RNA polymerase II as long primary transcripts (pri-miRNAs), which form a hairpin structure. In the nucleus, pri-miRNAs are recognized and processed by the microprocessor complex, composed of the RNase III enzyme Drosha–DGCR8, generating approximately 70-nt precursor miRNAs (pre-miRNAs) [62]. These precursor molecules are subsequently exported to the cytoplasm by Exportin-5 in a Ran-GTP-dependent manner. There, the pre-miRNA is processed by the RNase III endonuclease Dicer in association with the transactivation response RNA-binding protein (TRBP). Dicer cleaves the hairpin structure of the pre-miRNA to generate ~22 nucleotide miRNA duplex. The duplex is then loaded onto an Argonaute protein with the assistance of molecular chaperones, leading to the assembly of the RNA-induced silencing complex (RISC), where the mature miRNA guides sequence-specific recognition of target messenger RNAs, resulting predominantly in translational repression and/or mRNA destabilization depending on the degree of complementarity between the miRNA seed sequence and its target transcript. Through this mechanism, miRNAs act as fine regulators of gene expression, coordinating multiple signaling pathways rather than functioning as simple on/off molecular switches [63,64,65].

Although the canonical pathway accounts for the biogenesis of most mammalian miRNAs, several non-canonical pathways have also been described. Among these, mirtrons represent a distinct class of miRNAs derived from intronic sequences. Unlike canonical miRNAs, mirtron biogenesis bypasses Drosha-mediated processing and instead relies on spliceosome-mediated intron processing while remaining dependent on Dicer for maturation. Once incorporated into the RNA-induced silencing complex (RISC), mature mirtrons regulate gene expression through mechanisms analogous to those of canonical miRNAs, thereby contributing to the control of diverse cellular processes. Regardless of their biogenesis pathway, mature miRNAs ultimately associate with AGO proteins and regulate gene expression through similar post-transcriptional mechanisms [66,67,68].

While miRNAs were initially considered intracellular regulators acting exclusively within the cells in which they were produced, accumulating evidence has demonstrated that a subset of mature miRNAs is actively exported into the extracellular environment. These extracellular miRNAs remain remarkably stable despite the abundance of RNases in biological fluids because they are associated with protective carriers, including extracellular vesicles (EVs), Argonaute-containing ribonucleoprotein complex, high-density lipoproteins (HDL), and apoptotic bodies. Consequently, circulating miRNAs detected in plasma or serum represent a heterogeneous population derived from multiple cellular sources and transport mechanisms [16,18].

Throughout this review, EV nomenclature follows MISEV2023 [69]: we use “extracellular vesicles (EVs)” as the general term and “small EVs (sEVs)” or “medium/large EVs” as operational size descriptors. We reserve “exosome” and “ectosome” for EVs with demonstrated endosomal or plasma membrane origin, respectively; terminology from the original studies is identified explicitly when retained. In parallel, we distinguish total circulating miRNAs (measured in unfractionated plasma or serum), EV-associated miRNAs (measured in an EV-enriched fraction), and tumor-derived EV-miRNAs (assigned to a tumor source only when additional evidence supports that origin). Among these carriers, EVs have attracted considerable attention because they protect miRNAs from degradation while enabling their delivery to recipient cells. A fraction of mature miRNAs is selectively exported from the cytoplasm and packaged into EVs, particularly exosomes. This process, known as selective miRNA sorting, actively determines which miRNAs are incorporated into EVs and subsequently released into the extracellular environment. Rather than reflecting passive encapsulation of cytoplasmic RNA, selective miRNA sorting is a highly regulated biological process involving multiple molecular determinants [70].

Several RNA-binding proteins (RBPs), including Argonaute 2 (AGO2), hnRNPA2B1, and YBX1, recognize short sequence motifs that are overrepresented in specific miRNAs, known as EXOmotifs, thereby facilitating their selective incorporation into exosomes [71,72]. More recently, liquid–liquid phase separation (LLPS) has emerged as an additional mechanism contributing to miRNA sorting. In this process, the RNA-binding protein YBX1 undergoes phase separation to form biomolecular condensates that selectively concentrate specific miRNAs and facilitate their incorporation into nascent EVs [73]. Furthermore, post-transcriptional modifications of miRNAs, particularly 3′-end uridylation, enhance their interaction with sorting proteins and influence their preferential loading into EVs.

Importantly, the miRNA repertoire of EVs frequently differs from that of their donor cells, demonstrating that EV cargo is actively selected rather than representing a random sample of the intracellular transcriptome. Consequently, the molecular composition of EVs reflects regulated biological processes that may vary according to cell type, physiological state, and pathological conditions.

Following selective sorting, miRNAs can be released in EVs formed through endosomal or plasma membrane pathways, as well as in apoptotic bodies. Small EVs and medium/large EVs are operational size categories, not biogenesis-defined subtypes. Endosomal EVs are termed exosomes, whereas EVs budding directly from the plasma membrane are termed ectosomes. Because their size ranges overlap, these origins cannot be inferred from size alone; biogenesis-based terminology requires experimental evidence [69]. Endosome-derived small EVs, historically termed exosomes, remain the best-characterized carriers of regulatory miRNAs.

Exome biogenesis is a tightly regulated intracellular process that determines both vesicle composition and biological function. It begins with endocytosis of the plasma membrane, giving rise to early endosomes, which subsequently mature into late endosomes or multivesicular bodies (MVBs). During MVB maturation, the limiting endosomal membrane undergoes inward invagination to generate intraluminal vesicles (ILVs) that accumulate within the lumen of the MVB. The formation of ILVs and cargo incorporation are regulated either by the endosomal sorting complex required for transport (ESCRT) machinery or through ESCRT-independent mechanisms involving ceramide-, lipid-, and tetraspanin-mediated pathways [71,74,75]. These complementary mechanisms ensure selective cargo incorporation and contribute to the molecular heterogeneity of EVs.

MVBs have two principal fates: they may fuse with lysosomes, leading to degradation of the cargo, or traffic toward the plasma membrane for exosome secretion. The latter process is tightly regulated by Rab family GTPases, including Rab27a, Rab27b, Rab11, and Rab35, which coordinate vesicle trafficking, docking, and membrane fusion before exosome release [76,77]. Upon fusion of the MVB with the plasma membrane, the ILVs are released into the extracellular milieu as exosomes.

Once released into the extracellular milieu, EVs can interact with recipient cells, enabling the internalization and delivery of the miRNA cargo. Rather than occurring through passive diffusion, EV uptake is a tightly regulated process, and experimental evidence supports at least three major mechanisms of cellular internalization. (i) Endocytosis is considered the predominant uptake pathway and includes clathrin-mediated endocytosis, caveolin-mediated endocytosis, macropinocytosis, and phagocytosis. These processes are regulated by multiple cell-surface molecules, including tetraspanins, integrins, and other receptor-ligand interactions that contribute to EV recognition and cellular tropism [78,79]. (ii) Direct membrane fusion represents a less common mechanism in which the lipid bilayer of the EV fuses directly with the plasma membrane of the recipient cell, allowing the release of its luminal cargo into the cytoplasm [80,81]. (iii) Surface signaling occurs when EV membrane proteins interact with receptors on the recipient cell, triggering intracellular signaling cascades without requiring complete vesicle internalization or cargo release [81].

Following internalization, EVs enter the endosomal trafficking pathway, where they are first localized within early endosomes and subsequently sorted into MVBs. From this compartment, internalized EVs may undergo different intracellular fates. They can be recycled back to the plasma membrane and released into the extracellular environment or directed to lysosomes for degradation of both the vesicle membrane and its cargo [82]. Although the mechanisms governing endosomal escape remain incompletely understood, successful cytoplasmic delivery of EV-associated miRNAs is required for their incorporation into the RISC, enabling post-transcriptional regulation of target genes.

Importantly, not all extracellular miRNAs function as mediators of intracellular communication. A substantial proportion of circulating miRNAs is associated with AGO proteins, lipoproteins, or apoptotic material and primarily reflects physiological cell turnover or tissue injury rather than active extracellular signaling.

**Figure 2:**
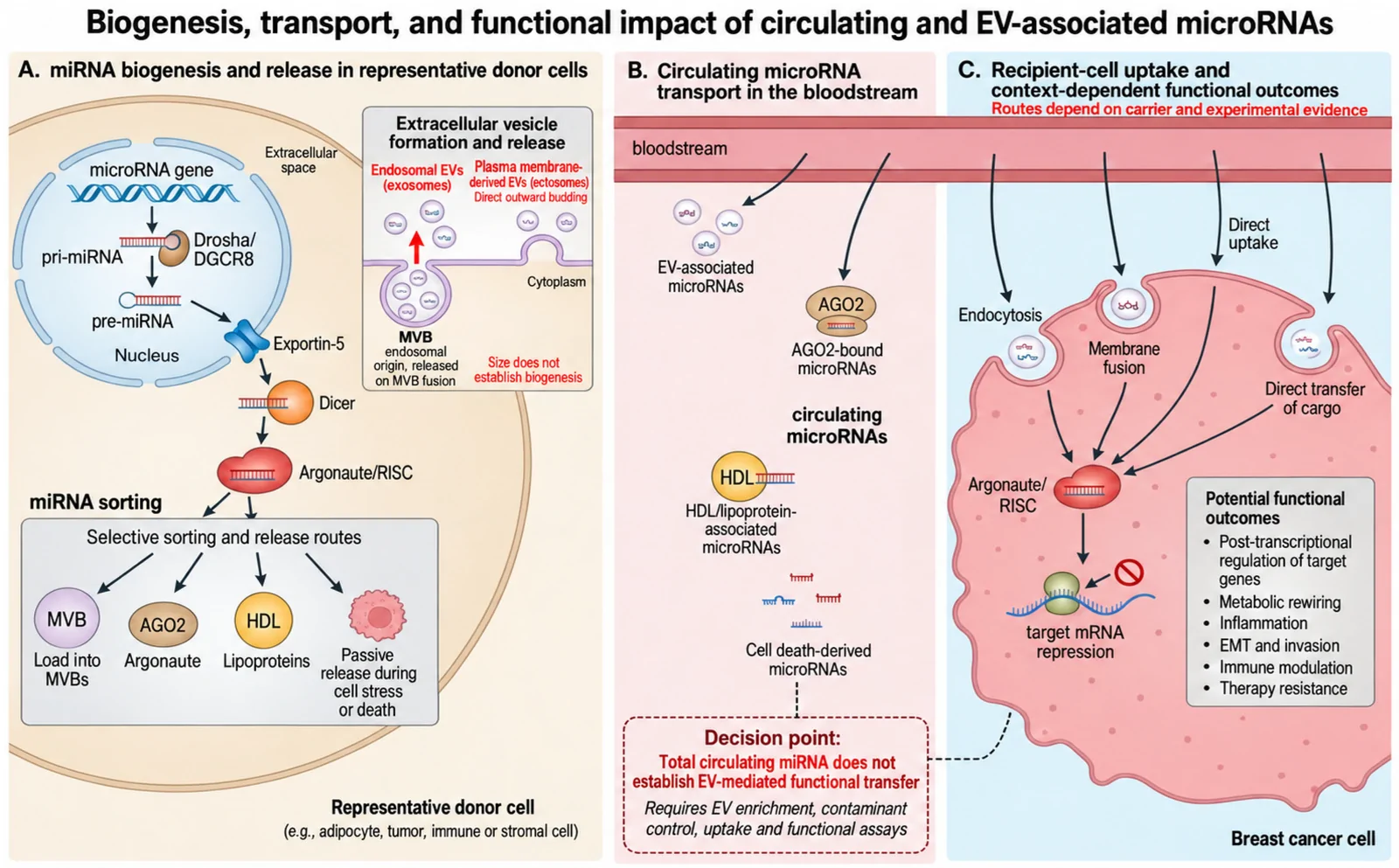
Biogenesis, transport, and functional impact of circulating and EV-associated microRNAs. (**A**) Canonical miRNA biogenesis begins with transcription of a microRNA gene into a pri-miRNA, followed by Drosha-/DGCR8-mediated processing in the nucleus, Exportin-5-dependent transport to the cytoplasm, Dicer-mediated maturation, and loading into Argonaute/RISC complexes. Mature miRNAs can then be selectively sorted into multivesicular bodies, associated with Argonaute proteins or lipoproteins, or passively released during cell stress or death. EV formation involves endosomal release of intraluminal vesicles following multivesicular body–plasma membrane fusion (exosomes) or direct outward budding from the plasma membrane (ectosomes). Small EVs and medium/large EVs are operational size categories; size alone does not establish biogenesis. (**B**) In the bloodstream, circulating miRNAs exist in different transport forms, including EV-associated miRNAs, AGO2-bound miRNAs, HDL/lipoprotein-associated miRNAs, and cell death-derived miRNAs. (**C**) Recipient breast cancer cells may internalize EV-associated or circulating miRNAs through endocytosis, membrane-mediated fusion, direct uptake, or direct transfer of cargo. Once inside recipient cells, miRNAs can be loaded into Argonaute/RISC complexes and repress target mRNAs, contributing to post-transcriptional regulation of gene expression and functional outcomes such as metabolic rewiring, inflammation, epithelial–mesenchymal transition, immune modulation, and therapy resistance. Decision point: total serum/plasma miRNA is not equivalent to EV-mediated functional transfer unless EV enrichment, contaminant control, uptake, and functional assays are shown. Red arrow indicates the release of endosomal-origin EVs through MVB fusion with the plasma membrane; the term “exosome” is reserved for EVs with experimentally demonstrated endosomal biogenesis.

In summary (Figure 2), selective miRNA sorting, EV biogenesis, regulated secretion, recipient-cell uptake, and functional cargo delivery together determine the biological impact of EV-associated miRNAs on intercellular communication. These processes are particularly relevant in obesity-associated breast cancer, where adipocytes, adipose-derived stromal cells, immune cells, endothelial cells, and tumor cells continuously exchange EVs, thereby reshaping the molecular landscape of the mammary tumor microenvironment. The contribution of obese adipose tissue as a source of circulating and EV-associated miRNAs is discussed in the following section.

### Circulating miRNAs as Biomarkers Versus EV-miRNAs as Functional Mediators

In this review, circulating miRNAs are interpreted under two complementary but distinct concepts. First, they may act as biomarkers, reflecting tumor burden, adipose tissue dysfunction, inflammation, platelet activation, endothelial stress, treatment exposure, or systemic metabolic disease. In this context, a circulating miRNA association does not imply causal activity. Second, a more restricted group of EV-associated miRNAs may act as functional mediators when experimental evidence demonstrates EV enrichment, uptake by recipient breast cancer cells, induction of a phenotype, and reversal or attenuation of that phenotype after miRNA inhibition, mimic manipulation, EV depletion, or pathway rescue.

For example, let-7a in serum EVs from overweight/obese breast cancer patients should currently be interpreted mainly as an obesity-stratified biomarker candidate, because the available evidence links its circulating EV expression with BMI and tumor grade, without demonstrating direct functional transfer or phenotype rescue; the reported survival association corresponds to tumor-level let-7a in public datasets rather than to serum EV measurements [83]. In contrast, breast adipose tissue-derived EVs from women with obesity have stronger EV-level functional support because they were shown to alter breast cancer cell proliferation, mitochondrial activity, oxidative phosphorylation-related programs, and Akt/mTOR-/P70S6K-related signaling. Their miRNA cargo includes obesity-associated candidates such as miR-155-5p, miR-10a-3p, and miR-30a-3p, although the individual causal contribution of each miRNA still requires direct gain/loss-of-function and rescue assays [37]. Similarly, EV-associated miR-221/222 has mediator-level evidence in endocrine resistance because transfer from tamoxifen-resistant breast cancer cells enhanced tamoxifen resistance in recipient ER-positive cells [84]. This distinction is essential to avoid assuming that all circulating miRNA changes are causal drivers of obesity-associated breast cancer.

To avoid overinterpretation, evidence is classified along two independent axes rather than along a single linear hierarchy, following current extracellular vesicle and biomarker reporting recommendations [85,86,87]. Axis 1, human relevance and source attribution, comprises four levels: (i) non-human or non-breast systems, including rodent models, differentiated adipocytes, and metabolic-disease models; (ii) human cells or human tissue explants; (iii) human biofluids, either from breast cancer patients or from cancer-free human donors used as an EV source in recipient-cell assays; and (iv) human breast cancer cohorts with explicit obesity or metabolic stratification. Axis 2, mechanistic causality, comprises four levels: (a) association only, that is, differential abundance without functional testing; (b) EV-level function, in which an intact EV preparation induces a phenotype in recipient cells; (c) miRNA-level function, requiring demonstrated uptake together with gain- or loss-of-function of the individual miRNA; and (d) causality with rescue, reserved for experiments in which the phenotype is reversed by an intervention directed at the miRNA itself, such as inhibition of that miRNA within the EV preparation or restoration of its target. Controls that act on the whole preparation or on a downstream pathway, such as EV depletion, blockade of a signaling pathway or metabolic inhibitors, support a mechanism of the intact EVs but do not identify the miRNA responsible, and are therefore reported separately rather than counted as level (d). When inhibitors are applied as a mix of several miRNAs, the evidence is annotated as (c–d) because causality is established for the set and not for each individual miRNA. Clinical validation is treated as a separate qualifier rather than as the top of a single scale, and is applied only when independent validation, prospective sampling where feasible, reported performance metrics, and adjustment for biological and pre-analytical confounders are available. The two axes are kept apart deliberately, because patient-derived evidence and in vivo mechanistic evidence are not interchangeable dimensions: serum EV let-7a is highly relevant to humans but association-only (iv/a), whereas adipocyte EV miR-27a in skeletal muscle is mechanistically informative but indirect for breast cancer (i/c). The same annotation is applied in Table 1 and in Supplementary Table S1.

## 4. Obese Adipose Tissue as a Source of Circulating and Extracellular Vesicle-Associated microRNAs

The recognition that adipose tissue functions as an endocrine organ capable of releasing EVs and regulatory miRNAs into the circulation has fundamentally changed our understanding of inter-organ communication. Rather than serving exclusively as an energy storage depot, adipose tissue actively exchanges molecular information with distant organs through the secretion of adipokines, lipids, metabolites, cytokines, and EV-associated nucleic acids. A landmark study by Thomou et al. demonstrated that adipose tissue contributes substantially to the pool of circulating EV-associated miRNAs (reported as exosomal), and adipose-derived miRNAs can regulate gene expression in distant tissues, establishing adipose tissue as an important component of systemic inter-organ communication [61]. These findings provide the conceptual basis for interpreting circulating miRNAs not only as tumor-derived biomarkers but also as host-derived mediators of systemic metabolic communication. Obesity profoundly remodels adipose tissue biology and consequently alters both the quantity and molecular composition of adipose-derived EVs. Adipocyte hypertrophy, chronic low-grade inflammation, hypoxia, extracellular matrix remodeling, oxidative stress, and metabolic dysfunction collectively modify EV biogenesis, secretion, and cargo selection [88], while these alterations contribute to adipose-tumor crosstalk in breast cancer [48,49].

Obesity can alter adipose EV production, molecular cargo, and biological activity through adipocyte hypertrophy, cellular stress, inflammation, and metabolic dysfunction. As indirect supporting evidence from non-breast metabolic models, experimental studies indicate that obesity-induced alterations in adipose tissue macrophage-derived EVs have been shown to regulate systemic insulin sensitivity by transferring specific miRNAs to metabolic target tissues. Likewise, adipocyte-derived small EV-associated miR-34a (reported as exosomal) promotes adipose inflammation by suppressing M2 macrophage polarization, thereby favoring a pro-inflammatory microenvironment. Similarly, adipocyte-derived small EV-associated miR-27a contributes to obesity-associated insulin resistance through modulation of PPARγ [88,89,90]. Although these studies were performed primarily in metabolic disease models rather than breast cancer, they provide compelling mechanistic evidence that obesity modifies EV-miRNA signaling through regulated changes in adipose tissue biology. Importantly, the obesity-associated EV signal is not restricted to miRNAs. Obesity increases the abundance of extracellular matrix protein 1 (ECM1) in circulating small EVs. Integrin-β2 governs the loading of ECM1 into the vesicles rather than its action on the recipient cell: its knockdown does not change cellular ECM1 levels but reduces ECM1 in the EVs released, and its overexpression has the opposite effect. The tumor-promoting consequences of this cargo were then demonstrated in murine models [22]. Adipose-derived EVs also carry proteins and lipids that engage TGF-β/SMAD signaling and fatty acid-dependent metabolic programs, contributing to epithelial–mesenchymal transition, matrix remodeling, and invasive behaviour independently of any single miRNA [21,91]. This has a direct methodological consequence for the framework used here: when an intact EV preparation induces a phenotype, the effect cannot be attributed to its miRNA cargo unless the individual miRNA is manipulated, which corresponds to level (c) or (d) of Axis 2.

This has significant implications for breast cancer studies. Circulating EV-miRNAs in patients with obesity may integrate signals from adipose tissue together with those derived from tumor, immune, endothelial, platelet, and other compartments. Thus, changes in circulating EV-miRNAs should not be interpreted as tumor-specific without additional evidence of their origin. Direct human evidence in obesity-associated breast cancer is illustrated by the study of Barone et al., in which serum EV-associated let-7a was reduced in overweight/obese breast cancer patients and inversely correlated with BMI, and lower let-7a discriminated high-grade tumors; the reported survival association derives from tumor-level let-7a in public datasets rather than from circulating EV measurements [83]. These findings support its consideration as an obesity-stratified biomarker candidate but do not demonstrate adipose origin or a direct effect on tumor cells. The influence of metabolic status on circulating miRNAs is further supported by studies showing changes in circulating miRNA profiles after weight-loss interventions in women with overweight or obesity [92]. Taken together, these observations indicate that obesity is an important biological determinant of the circulating EV-miRNA landscape. Representative circulating and EV-associated miRNAs relevant to obesity-associated breast cancer are summarized in Table 1.

**Figure 3:**
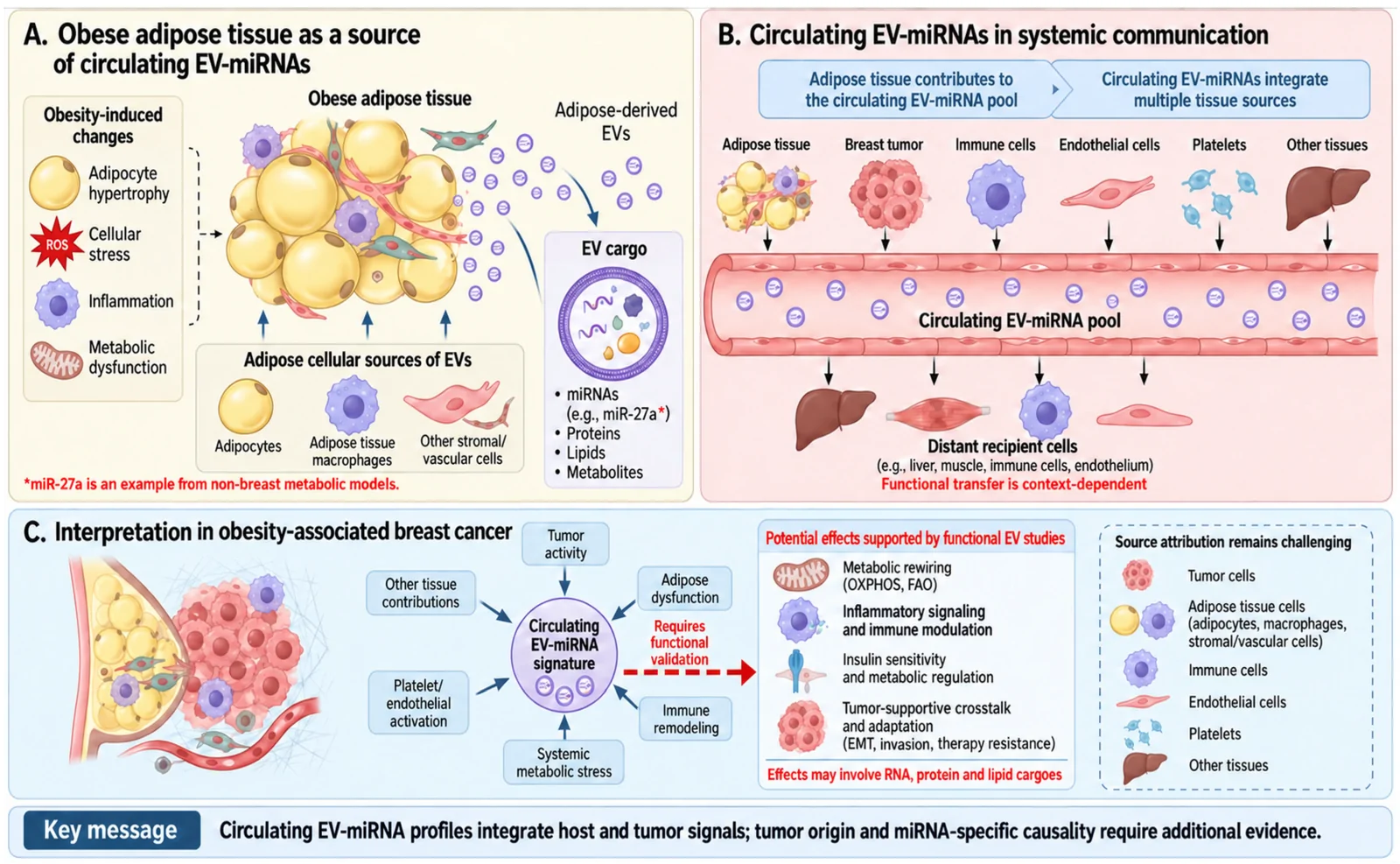
Obese adipose tissue as a source of circulating extracellular vesicle-associated microRNAs and its implications for biomarker interpretation. (**A**) Obesity alters adipose tissue through adipocyte hypertrophy, cellular stress, inflammation, and metabolic dysfunction, thereby modifying the release and molecular cargo of adipose-derived EVs. Adipocytes, adipose tissue macrophages, and other stromal and vascular cells contribute to the adipose EV pool, which contains miRNAs together with proteins, lipids, and metabolites. (**B**) Adipose-derived EVs contribute to the circulating EV-miRNA pool and may participate in systemic inter-organ communication. (**C**) In breast cancer, however, circulating EV-miRNA profiles represent an integrated host–tumor signal that may reflect tumor activity, adipose dysfunction, immune and endothelial remodeling, platelet activation, and systemic metabolic stress. Therefore, circulating EV-miRNA signatures should not be interpreted as exclusively tumor-derived biomarkers without evidence supporting their cellular or tissue origin. Arrows indicate conceptual directionality rather than experimentally established lineage. Blue arrows denote contributions of cellular or tissue sources to EV release, EV cargo, or the circulating EV-miRNA signature. Black arrows indicate the directional flow of EV-associated miRNAs from donor tissues into the circulation and toward potential recipient cells. The red dashed arrow indicates that linking a circulating EV-miRNA signature to specific downstream biological effects requires functional validation and should not be interpreted as established causality.

Taken together, these observations indicate that obesity is an important biological determinant of the circulating EV-miRNA landscape. The contribution of obese adipose tissue to the circulating EV-miRNA pool and its implications for biomarker interpretation are summarized in Figure 3. The potential biological consequences of adipose-derived EV communication at the breast tumor interface are discussed in the following section.

**Table 1:** Representative circulating and extracellular vesicle-associated miRNAs implicated in obesity-associated breast cancer: current evidence and biological interpretation.

miRNA	Principal Source of Evidence	Main Biological Function	Evidence in Obesity-Associated Breast Cancer	Level of Evidence	References
Human cohort; association only (iv/a). Clinical biomarker candidate.					
let-7a-5p	Serum EVs from breast cancer patients stratified by BMI	Tumor-suppressor; metabolic regulation	Downregulated in serum EVs from overweight/obese breast cancer patients and inversely associated with BMI and with higher tumor grade. The reported association with survival corresponds to tumor-level let-7a in public datasets, not to serum EV let-7a. Current evidence supports its role primarily as an obesity-stratified EV biomarker rather than a demonstrated functional mediator.	Human cohort; association only (iv/a). Clinical biomarker candidate.	[83]
miR-122-5p	Serum EV profiling in obesity-stratified breast cancer; tumor-derived EVs.	Metabolic regulation; premetastatic niche formation.	Differentially expressed in obesity-stratified breast cancer EVs. Interpretation requires caution because miR-122-5p also participates in hepatic metabolism and tumor-derived EV communication.	Human cohort; association only (iv/a). Clinical biomarker candidate.	[83,93]
miR-126-3p	Circulating/EV-associated miRNA; endothelial compartment	Angiogenesis and vascular remodeling	May reflect endothelial dysfunction microenvironment remodeling rather than tumor-specific signaling.	Human cohort; association only (iv/a). Clinical biomarker candidate.	[83,94]
miR-21	Circulating and EV-associated miRNA in breast cancer	OncomiR involved in proliferation, invasion, inflammation and therapy resistance.	Frequently proposed as a breast cancer biomarker but not obesity specific. Circulating levels may reflect tumor burden, systemic inflammation, stromal remodeling, or metabolic dysfunction.	Human biofluids from breast cancer patients, with age- and BMI-matched controls but without obesity stratification; association only (iii/a). Clinical biomarker candidate.	[95,96]
Functional adipose-derived EV-miRNAs					
miR-155-5p	Breast adipose tissue-derived EVs from women with obesity.	Immunometabolic signaling; proliferation; mitochondrial regulation.	Enriched in obesity-associated breast adipose EVs. A miR-155-5p mimic increased both proliferation and mitochondrial respiration in recipient cells, and inhibition of this miRNA within the EVs, as part of a three-miRNA inhibitor mix, reduced the respiratory parameters induced by the O-EVs.	Human adipose tissue EVs; EV-level function plus miRNA-level gain of function and inhibition (ii/c–d). Pre-incubation of the O-EVs with a mix of LNA inhibitors against miR-155-5p, miR-10a-3p and miR-30a-3p reduced basal respiration, ATP production and maximal respiratory capacity, and reduced proliferation in a representative experiment, although across seven EV cases the effect on proliferation was not significant. Because the inhibitors were applied as a mix, the contribution of each individual miRNA is not isolated.	[37,97].
miR-10a-3p	Breast adipose tissue-derived EVs from women with obesity	Metabolic reprogramming; oxidative phosphorylation.	Enriched in breast adipose EVs from women with obesity. A miR-10a-3p mimic significantly increased basal respiration, ATP production and maximal respiratory capacity in recipient cells, without a significant effect on proliferation; it was also included in the inhibitor mix that reduced the respiratory response to O-EVs.	Human adipose tissue EVs; EV-level function plus miRNA-level gain of function and inhibition within the EVs, applied as a three-miRNA mix (ii/c–d).	[37]
miR-30a-3p	Breast adipose tissue-derived EVs from women with obesity	Cell proliferation; mitochondrial function.	Identified among obesity-associated breast adipose EV miRNAs. A miR-30a-3p mimic significantly stimulated proliferation of recipient cells, whereas its effect on mitochondrial respiration was not significant; it was also included in the inhibitor mix applied to the O-EVs.	Human adipose tissue EVs; EV-level function plus miRNA-level gain of function and inhibition within the EVs, applied as a three-miRNA mix (ii/c–d).	[37]
miR-34a	Adipocyte-derived small EVs (reported as exosomes).	Macrophage polarization; adipose inflammation.	Promotes obesity-associated adipose inflammation by inhibiting M2 macrophage polarization. Its relevance to breast cancer is indirect and likely mediated through remodeling of the inflammatory adipose microenvironment.	Non-breast metabolic model; miRNA-level function with rescue (i/d): adipocyte-specific deletion of miR-34a and reversal of the phenotype by ectopic KLF4 expression in recipient macrophages.	[89]
miR-27a	Adipocyte-derived small EVs (reported as exosomes).	PPARγ signaling; insulin resistance.	Demonstrates that adipocyte-derived EV-miRNAs regulate systemic metabolic dysfunction. Its relevance to breast cancer is indirect and should be interpreted in the context of obesity-associated insulin resistance and metabolic stress.	Non-breast metabolic model; miRNA-level function (i/c).	[90]
Tumor-derived EV-miRNAs					
miR-221/222	Small EVs from endocrine-resistant breast cancer cells (reported as exosomes).	Endocrine resistance; PTEN/p27 signaling	Experimental evidence demonstrates EV-mediated transfer of miR-221/222 from tamoxifen-resistant to sensitive breast cancer cells, promoting endocrine resistance. Although not obesity-specific, this pathway may become particularly relevant when obesity-associated metabolic dysfunction coexists with endocrine therapy.	Breast cancer cell models; miRNA-level function, not obesity-stratified (ii/c). Reference [83] supports experimental EV-mediated transfer, whereas reference [90] is a separate clinical study of circulating plasma miR-221/222 that does not test EV-mediated transfer (iii/a).	[84,98]

Table note: EV, extracellular vesicle; PPARγ, peroxisome proliferator-activated receptor γ; PTEN, phosphatase and tensin homolog. This table prioritizes representative miRNAs with direct or indirect relevance to obesity-associated breast cancer, adipose EV-mediated metabolic communication, or breast cancer EV biology. Evidence is annotated with the two-axis framework defined in Section 3. Axis 1, human relevance and source attribution: (i) non-human or non-breast systems; (ii) human cells or tissue explants; (iii) human biofluids, from breast cancer patients or from cancer-free donors used as an EV source; (iv) human breast cancer cohorts with obesity or metabolic stratification. Axis 2, mechanistic causality: (a) association only; (b) EV-level function; (c) miRNA-level function; (d) causality with rescue directed at the miRNA itself. Clinical validation is a separate qualifier. Inclusion does not imply that each miRNA is a validated causal mediator. The mechanistic relationships of EV-associated miRNAs and cargoes at the obesity–breast cancer interface are summarized in Supplementary Table S1.

## 5. Extracellular Vesicle-Associated miRNAs at the Interface of Obese Adipose Tissue and the Breast Tumor Microenvironment

**Figure 4:**
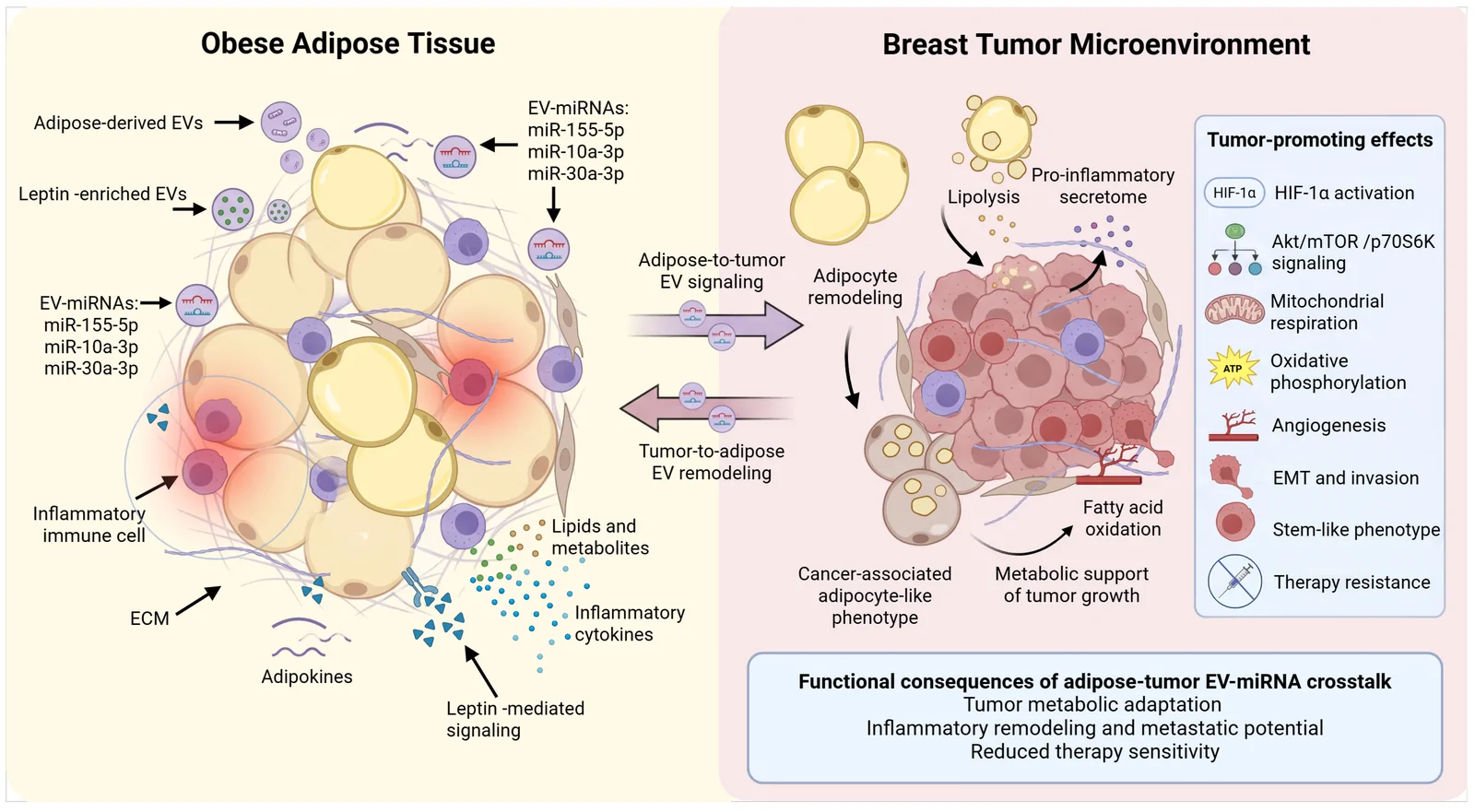
Bidirectional communication between obese adipose tissue and breast cancer cells. Obese adipose tissue-derived extracellular vesicles deliver miRNAs, proteins, lipids, leptin-related signals, and metabolic cargoes to breast cancer cells. These signals may enhance oxidative phosphorylation, fatty acid oxidation, hypoxia-inducible factor 1-alpha activation, Akt/mTOR signaling, migration, invasion, epithelial–mesenchymal transition, and therapy resistance. Conversely, tumor-derived extracellular vesicles remodel adipocytes, immune cells, fibroblasts, and stromal cells toward a tumor-permissive phenotype. Evidence label: Human adipose/breast-adipose EV data should be separated from preclinical adipocyte or tumor-cell EV models. Curved black arrows indicate proposed local interactions and remodeling processes within the tissue microenvironment rather than a discrete experimentally traced transfer route.

The breast tumor microenvironment is closely connected to surrounding adipose tissue. Obesity promotes adipocyte hypertrophy, inflammation, metabolic stress, and extracellular matrix remodeling, creating conditions that may influence tumor behavior. In addition to soluble mediators, extracellular vesicles provide a mechanism for the exchange of molecular cargo between adipose tissue and breast cancer cells. This communication is bidirectional, as illustrated in Figure 4.

Human tissue-based evidence provides the strongest support for obesity-associated adipose-tumor EV communication. Liu et al. showed that EVs derived from breast adipose tissue of women with overweight or obesity increased breast cancer cell proliferation, mitochondrial density, oxidative phosphorylation-related gene expression, and mitochondrial respiration. These EVs were enriched in miR-155-5p, miR-10a-3p, and miR-30a-3p, and their effects were associated with Akt/mTOR/P70S6K signaling [37]. These findings provide functional evidence for adipose-derived EV effects on cancer cells, although the specific contribution of each miRNA remains to be established.

Other studies support a broader role for adipose-derived EVs in tumor progression. Adipocyte-derived EVs have been associated with HIF-1α activation, growth, migration, invasion, EMT, and stem-like phenotypes [99]. EVs derived from adipocytes or from overweight/obese breast cancer patients have also been reported to increase mitochondrial activity, ATP production, oxygen consumption, and oxidative phosphorylation in estrogen receptor-positive breast cancer cells [100]. Within this human tissue-based evidence, the individual miRNAs were also tested directly. Recipient cells transfected with mimics reproduced part of the phenotype: miR-155-5p increased both proliferation and mitochondrial respiration, miR-10a-3p increased respiration without a significant effect on proliferation, and miR-30a-3p increased proliferation without a significant effect on respiration. Conversely, pre-incubation of the vesicles with LNA inhibitors directed against the three miRNAs reduced basal respiration, ATP production and maximal respiratory capacity of the educated cells, and reduced proliferation in the representative experiment, although across seven independent EV preparations the reduction in proliferation did not reach significance. Because the inhibitors were applied as a mix, this combined loss of function supports a contribution of the group of miRNAs to the respiratory phenotype without resolving the individual contribution of each one. Beyond this, these studies demonstrate biological effects of EVs as a whole and do not establish that miRNAs are solely responsible for the responses observed, since part of the effect is attributable to protein and lipid cargoes acting through TGF-β/SMAD and fatty acid-dependent programs [21,22]. The metabolic phenotype of the donor, rather than body mass index alone, also appears to determine the biological activity of these vesicles: In diet-induced obese mice, plasma and adipocyte EVs induced epithelial–mesenchymal transition features and increased migration in vitro and metastasis in vivo in syngeneic triple-negative tumor cells, indicating that the metabolic state of the donor defines a functionally distinct EV population and supporting the metabolic phenotyping recommended in Section 9. This evidence is entirely murine [23]. Consistent with this, EVs isolated from the adipose tissue secretome of individuals with obesity carry elevated TGF-β and activate TGF-β/SMAD signaling in MCF-7 cells, reducing E-cadherin expression and increasing migration and invasiveness; these EVs are also enriched in fatty acids that fuel the tumor cells through fatty acid oxidation [91]. This interaction is not unidirectional: breast cancer cells can release EVs that promote adipocyte remodeling, lipolysis, inflammatory responses, and metabolic changes, potentially increasing the availability of fatty acids and other nutrients within the tumor microenvironment. The resulting adipose phenotype may, in turn, reinforce the local availability of metabolites and pro-tumoral signals [48,101].

Overall, current evidence supports a model in which EVs participate in reciprocal adipose–tumor communication and contribute to metabolic adaptation, inflammatory remodeling, invasion, and other tumor-promoting phenotypes. However, a distinction should be maintained between functional effects of intact EVs and causal effects of individual EV-associated miRNAs. At present, the evidence for the former is stronger than for the latter, particularly in human obesity-associated breast cancer. This distinction is important when interpreting miRNAs such as miR-155-5p, miR-10a-3p, and miR-30a-3p, which are enriched in EVs from obese breast adipose tissue but have not yet been individually validated as the principal mediators of the observed tumor-cell phenotype.

Together, these findings are consistent with a broader view in which adipose tissue-derived EVs participate in several cancer-related programs, including metabolic rewiring, inflammatory signaling, angiogenesis, extracellular matrix remodeling, invasion, and therapy resistance. Reviews focused on adipose tissue-derived EVs and the tumor microenvironment have proposed that obesity may amplify these vesicular signals by increasing adipocyte stress, immune-cell infiltration, altered adipokine secretion, and the release of EV cargoes enriched in regulatory RNAs, proteins, lipids, and metabolites [101,102,103]. In the breast tumor microenvironment, EV-miRNA-mediated communication is especially relevant because tumor cells, adipocytes, adipose-derived stromal cells, endothelial cells, fibroblasts, and immune cells can exchange miRNA cargo that modulates angiogenesis, invasion, stromal remodeling, immune responses, and treatment adaptation [20]. Thus, obesity-associated breast adipose EVs should be interpreted not only as isolated miRNA carriers, but as part of a broader vesicular communication system that integrates metabolic stress, adipose dysfunction, and tumor cell adaptation.

Taken together, the hypoxia- and metabolism-related effects described above support metabolic adaptation and potential therapeutic tolerance, but they remain EV-level observations and should be kept separate from purely biomarker-level associations [99,100].

Metabolic comorbidities add another layer of complexity to adipose-tumor EV communication. In the mechanistic context, systemic EVs from women with obesity support the concept that host-derived vesicles can increase invasive behavior and extracellular matrix-remodeling activity in breast cancer cells [104]. The endocrine therapy implications of this finding are discussed in Section 7, where they can be distinguished from tumor-derived EV transfer of resistance traits.

Evidence from obesity-associated triple-negative breast cancer further suggests that host metabolic background, race, and tumor subtype may shape miRNA-mRNA regulatory networks and tumor biology. For this reason, candidate miRNAs should be annotated by tumor subtype and metabolic context rather than presented as universal breast cancer biomarkers. When evidence is limited to differential expression or network analysis, it should be explicitly labeled as associative and hypothesis-generating.

A remaining challenge is to determine which fraction of circulating EV-miRNAs originates from tumor cells versus adipose or stromal compartments. Proteomic and surface-marker approaches may help enrich specific EV subpopulations, but marker interpretation requires caution because many proposed EV markers are not exclusive to a single tissue or cell type. For adipocyte-derived EVs, markers such as adiponectin, FABP4, or PLIN1 have been discussed, although PLIN1 may also reflect lipid droplet-associated material and potential co-isolated artifacts rather than a universally validated EV surface marker. Therefore, source attribution should ideally combine EV characterization, proteomic profiling, matched tumor–adipose tissue–plasma analysis, small RNA sequencing, and functional validation in recipient cells [105,106].

To integrate the mechanistic evidence discussed above, Supplementary Table S1 summarizes representative circulating and EV-associated miRNAs according to the proposed pathway, metabolic node, breast cancer phenotype, model or biological source, and level of mechanistic support (Figure 4).

## 6. Diagnostic and Prognostic Potential of Circulating miRNAs in Breast Cancer

Circulating miRNAs have been extensively studied as non-invasive biomarkers for breast cancer diagnosis, prognosis, recurrence prediction, and therapy monitoring. Their stability in blood, detectability by quantitative reverse transcription polymerase chain reaction, droplet digital polymerase chain reaction, microarrays, and small RNA sequencing, and their association with tumor biology make them attractive candidates for liquid biopsy approaches. Molecular liquid biopsy readouts may also be combined with cell-level biophysical characterization, such as three-dimensional electrophoretic and dielectrophoretic profiling of cancer cells, which provides complementary information on invasive behavior but does not measure circulating miRNAs and is therefore best regarded as an orthogonal, not an alternative, diagnostic layer [107]. Large-cohort studies have shown that combined serum miRNA panels may improve early breast cancer detection compared with single-marker approaches [108], whereas studies across heterogeneous recruitment sites indicate that circulating miRNA signatures may lose performance when applied to populations with different genetic, environmental, or clinical backgrounds [109]. Therefore, diagnostic and prognostic miRNA signatures require external validation before clinical translation.

Circulating miRNA models have also been explored for therapy-response prediction in metastatic breast cancer, supporting their potential use in treatment monitoring, although these applications remain investigational and require prospective validation [110].

For clinical biomarker interpretation, circulating miRNA evidence should be reported using a standardized and operational structure: sample type, anticoagulant and processing conditions, hemolysis control, platelet depletion or residual platelet assessment, RNA isolation method, spike-ins and endogenous normalization strategy, detection platform, cohort size, tumor subtype, obesity/metabolic stratification, diagnostic or prognostic performance metrics, and external validation, in line with STARD and REMARK recommendations [110,111] (Table 2). This is especially important because miRNAs such as miR-21 and miR-155 are not tumor-specific by default and may also reflect inflammation, immune activation, stromal remodeling, platelet or endothelial signals, diabetes, medication exposure, and obesity-associated metabolic dysfunction [112,113,114].

For example, a recent meta-analysis reported high diagnostic performance for circulating miR-155 in breast cancer, but the estimates differed by sample type. Serum miR-155 showed higher diagnostic performance than plasma miR-155, with reported sensitivity of 0.94, specificity of 0.89, and AUC of 0.97 for serum, compared with sensitivity of 0.87, specificity of 0.72, and AUC of 0.73 for plasma [115]. These findings support miR-155 as a promising diagnostic candidate, but they also illustrate why sample type, pre-analytical handling, tumor subtype, metabolic phenotype, and validation design must be reported explicitly. Representative human studies of circulating and EV-associated miRNAs in breast cancer are summarized in Table 3, annotated by cohort, sample type and fraction, tumor subtype, obesity or metabolic stratification, analytical platform, reported performance, and independent validation; studies that were not stratified by obesity are identified as such, because they inform breast cancer biomarker development in general rather than the obesity-associated setting addressed here. Therefore, diagnostic, prognostic, or predictive claims should remain framed as candidate or investigational unless supported by independent validation, robust performance metrics, and adjustment for metabolic and pre-analytical variables.

## 7. Circulating miRNAs and Implications in Breast Cancer Therapy Response

Therapy resistance in breast cancer is a multifactorial process involving tumor-intrinsic alterations, microenvironmental signals, immune remodeling, metabolic adaptation, and vesicular communication. miRNAs can influence response to endocrine therapy, chemotherapy, HER2-targeted therapy, immunotherapy, and radiation by modulating apoptosis, autophagy, DNA repair, epithelial–mesenchymal transition, stemness, drug efflux, immune checkpoints, and oncogenic signaling pathways [126,127,128]. In parallel, extracellular vesicles can transfer proteins, miRNAs, lncRNAs, circRNAs, mitochondrial DNA, and other regulatory cargoes between resistant and sensitive cells, contributing to therapy adaptation within the breast tumor microenvironment [42,129,130].

Endocrine resistance is especially relevant in obesity-associated breast cancer because estrogen receptor-positive tumors are common in postmenopausal women, and obesity can increase estrogen production, insulin/IGF signaling, leptin signaling, inflammatory cytokines, and PI3K/AKT/mTOR pathway activation. Small EV-associated miR-221/222 (reported as exosomal) remains a useful mediator-level example because vesicles from tamoxifen-resistant cells can enhance tamoxifen resistance in recipient estrogen receptor-positive breast cancer cells [84]. However, this tumor-derived resistance-transfer mechanism should not be considered obesity-specific unless metabolic stratification is included in the study design.

Obesity-associated circulating small EVs may also modify therapy response through host metabolic conditioning. Plasma-derived small EVs (reported as exosomes) from women with obesity promoted proliferation, migration, invasion, matrix metalloproteinase activity, and tamoxifen resistance in MCF-7 cells [104]. This provides EV-level support for host conditioning, obtained with EVs from cancer-free human donors with obesity applied to MCF-7 cells rather than from breast cancer patients, but clinical application still requires prospective treatment-stratified cohorts, tumor subtype annotation, metabolic phenotyping, and standardized EV-miRNA workflows.

Metabolic rewiring may further contribute to therapy tolerance. Adipocyte-derived EVs and EVs from overweight/obese patients have been linked to HIF-1α activity, mitochondrial metabolism, oxidative phosphorylation, invasion, epithelial–mesenchymal transition, and stem-like traits in breast cancer cells [99,100]. In the therapeutic context, these adaptations may provide energetic flexibility under endocrine or cytotoxic stress, although direct prospective evidence linking obesity-associated EV-miRNA profiles with treatment outcome remains limited.

**Figure 5:**
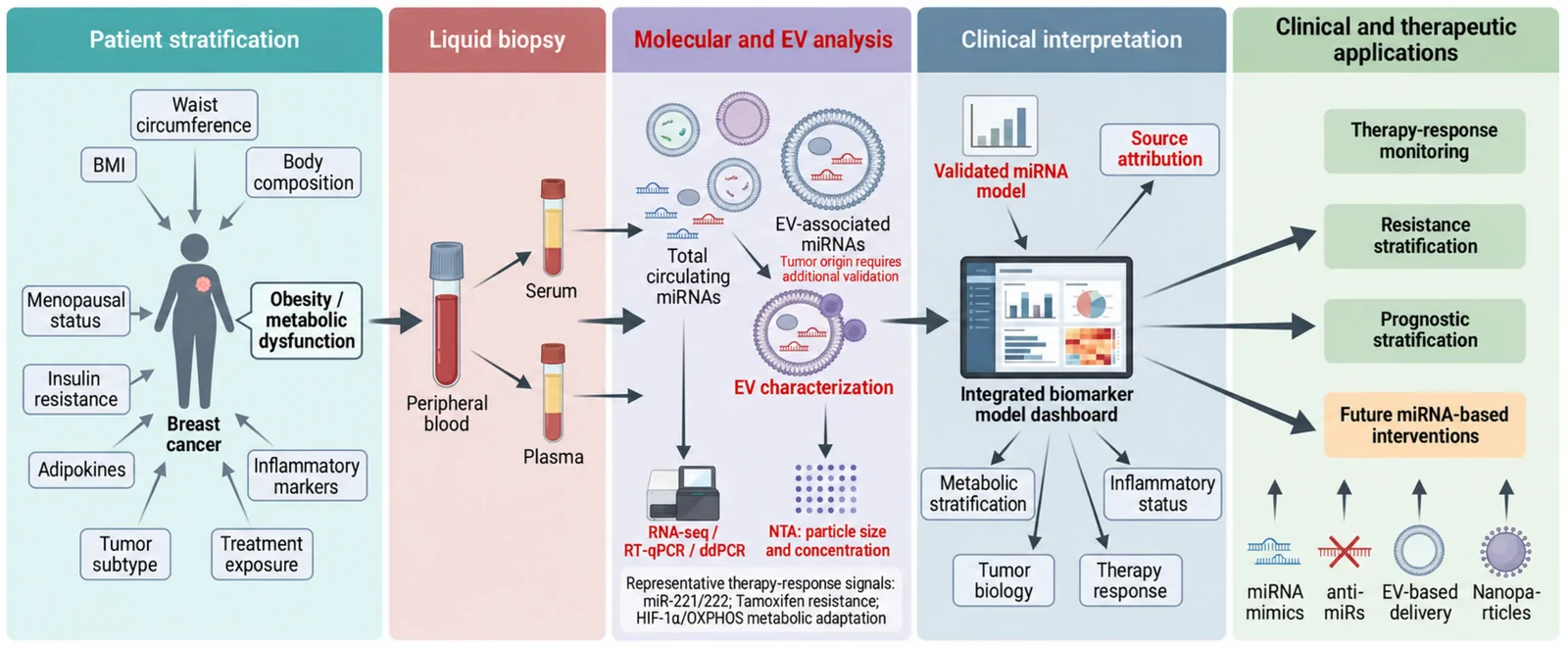
Proposed translational research workflow of circulating microRNAs in obesity-associated breast cancer therapy response. Patient stratification should incorporate metabolic and clinical variables such as body mass index, waist circumference, body composition, menopausal status, insulin resistance, adipokines, inflammatory markers, tumor subtype, and treatment exposure. Peripheral blood-derived serum and plasma can then be used for liquid biopsy-based analysis of total circulating miRNAs and EV-associated miRNAs. These data may be integrated using RNA-seq, nanoparticle tracking analysis, and qRT-PCR/ddPCR-based models to generate biomarker frameworks linked to metabolic stratification, tumor biology, inflammatory status, and therapy response. In obesity-associated breast cancer, this approach may support therapy-response monitoring, resistance stratification, prognostic evaluation, and the future development of miRNA-based interventions, including miRNA mimics, anti-miRs, EV-based delivery, nanoparticles, and metabolically informed therapeutic strategies. Decision point: distinguish tumor-derived EV resistance transfer from host metabolic EV conditioning before proposing a therapeutic mechanism.

In triple-negative and HER2-positive breast cancer, EV-associated non-coding RNAs have also been implicated in chemoresistance, immune escape, epithelial–mesenchymal transition, metastatic progression, and targeted therapy resistance. Reviews focused on EV-mediated chemoresistance and breast cancer progression highlight that EV cargoes can transmit drug-resistant phenotypes and may serve as minimally invasive markers of treatment response [131,132,133]. However, in obesity-associated breast cancer, such signals should be interpreted together with metabolic stratification, tumor subtype, menopausal status, inflammation, and treatment exposure. A translational workflow integrating circulating miRNAs, metabolic phenotyping, treatment monitoring, and future miRNA-based interventions is proposed in Figure 5.

### Interpretative Note: Tumor-Derived EV Resistance Transfer Versus Obesity-Associated Host EV Effects

A key conceptual distinction is the difference between tumor-derived EV-mediated resistance transfer and obesity-associated host EV conditioning. Tumor-derived EV resistance transfer refers to vesicles released by resistant cancer cells that horizontally transmit resistance-associated cargo, as shown for small EV-associated (reported as exosomal) miR-221/222 in tamoxifen-resistant breast cancer models [84]. Obesity-associated host EV conditioning refers to plasma-, adipose tissue-, or adipocyte-derived EVs from an obese systemic or mammary environment that alter tumor metabolism, invasion, or endocrine sensitivity [37,99,104]. Keeping these mechanisms separate prevents overinterpretation and makes therapy-response claims more operational.

## 8. Therapeutic Perspectives: Targeting miRNA Networks in Obesity and Breast Cancer

miRNA-based therapeutics offer the possibility of modulating regulatory networks rather than single molecular targets. In cancer, two main strategies have been explored: restoration of tumor-suppressive miRNAs using miRNA mimics and inhibition of oncogenic miRNAs using antagomiRs, antisense oligonucleotides, locked nucleic acid-modified inhibitors, sponges, or nanoparticle-based delivery systems. In breast cancer, these approaches remain largely preclinical, but they continue to attract interest because miRNAs may simultaneously influence proliferation, invasion, metastasis, angiogenesis, immune escape, stemness, and therapy resistance [134,135,136]. However, clinical translation remains limited by challenges related to target specificity, off-target effects, immune activation, RNA stability, tissue delivery, dosing, toxicity, and tumor heterogeneity [137,138].

It should be stated explicitly that no miRNA-directed intervention has been clinically validated in breast cancer, and that none has been evaluated in obesity-associated breast cancer as a defined clinical setting: the available evidence is preclinical, and the few miRNA therapeutics that reached early-phase oncology trials, such as the miR-34a mimic MRX34 [139], were tested in advanced solid tumors and enrolled only isolated breast cancer cases, without demonstrating efficacy in breast cancer and without any metabolic stratification. Engineered EV-based delivery is at a comparable stage; the KRAS-targeted engineered EV programme illustrates both the feasibility of the approach and the distance that still separates it from routine oncology practice [140]. In obesity-associated breast cancer, a therapeutic framework should not focus only on tumor cells. A broader strategy would aim to interrupt the adipose–tumor communication network. Potential approaches include reducing adipose inflammation, restoring tumor-suppressive miRNAs such as members of the let-7 family, inhibiting oncogenic or inflammatory miRNAs, blocking pathological EV release or uptake, and disrupting adipokine-, HIF-1α-, and Akt/mTOR-related metabolic adaptation. This concept is supported by evidence that obesity-associated adipose EVs can alter tumor cell metabolism and oxidative phosphorylation-related programs, and that adipocyte-derived EVs can promote HIF-1α-dependent malignant phenotypes in breast cancer cells [37,99,141]. Therefore, therapeutic targeting of miRNA networks in this context should integrate oncology, metabolism, and microenvironmental biology rather than treating circulating miRNAs as tumor-only molecules.

Extracellular vesicles themselves may also be developed as delivery vehicles for therapeutic RNAs. Their lipid bilayer can protect RNA cargo, and their natural ability to interact with recipient cells makes them attractive for miRNA mimic, anti-miR, and small RNA delivery. Nevertheless, the therapeutic use of engineered EVs in breast cancer remains mostly preclinical and must still resolve scalability, cargo loading, biodistribution, off-target delivery, immune activation, safety, and reproducible potency assays before clinical translation [129,134,142,143,144,145,146].

An additional therapeutic opportunity is the indirect modulation of circulating and EV-associated miRNAs through metabolic interventions. Weight loss, exercise, bariatric surgery, dietary modification, insulin-sensitizing approaches, and anti-inflammatory strategies may remodel the circulating miRNA landscape, but these interventions should be interpreted as systemic metabolic modulators rather than direct anti-miRNA therapies unless paired molecular and clinical response data are available [92].

## 9. Methodological Challenges and Future Directions

The clinical translation of circulating and EV-associated miRNAs in obesity-associated breast cancer remains limited by methodological heterogeneity and by the difficulty of distinguishing tumor-related signals from host-derived changes. Rather than focusing primarily on the discovery of additional candidate miRNAs, future studies should prioritize sample handling, reliable EV characterization, appropriate metabolic phenotyping, source attribution, and independent validation.

Circulating miRNA measurements are highly sensitive to pre-analytical conditions. Studies should therefore report the type of biofluid analyzed, fasting status, time from blood collection to processing, centrifugation protocol, storage conditions, and freeze–thaw history. Hemolysis and residual platelet contamination should also be assessed because erythrocytes and platelets contain abundant miRNAs that can substantially influence circulating profiles. RNA extraction procedures, spike-in controls, and normalization strategies should be described in sufficient detail to allow comparison between studies.

For EV-focused studies, these considerations are particularly important because the measured signal depends on how EVs are separated from other circulating components. MISEV2018 and MISEV2023 provide a framework for reporting EV studies, including the separation method, particle concentration and size distribution, morphological or orthogonal characterization, positive and negative EV markers, and assessment of potential contaminants [85,121]. Studies should also clearly indicate whether they measure total circulating miRNAs, EV-enriched miRNAs, or miRNAs associated with a defined EV subpopulation. Without this information, EV-miRNA measurements may include AGO-bound RNA, lipoprotein-associated miRNAs, platelet-derived RNA, or RNA released during cell death.

Normalization remains another major source of variability, because commonly used extracellular controls, including U6, are not necessarily stable in plasma or serum and should not be used without prior validation in the biological matrix being studied. Hemolysis assessment and normalization strategies should therefore be selected according to the sample type and experimental design rather than applied uniformly across studies [116,117,118,120,147]. The analytical platform also matters: stem-loop RT-qPCR, polyadenylation-based methods, adapter ligation approaches, and targeted assays differ in sensitivity, specificity, throughput, and their ability to distinguish closely related miRNAs and isomiRs. These differences become particularly relevant when circulating miRNAs are present at low abundance or when samples contain variable amounts of hemolysis- or platelet-derived RNA [148].

BMI alone provides an incomplete description of the metabolic environment in which circulating miRNAs are measured. Future studies should, whenever possible, incorporate waist circumference (WC), waist-to-hip ratio (WHR), visceral and subcutaneous adiposity, menopausal status, diabetes or insulin resistance, circulating adipokines, inflammatory markers, medication use, and treatment status. These variables may influence circulating miRNA abundance independently of tumor burden and may therefore act as important confounders in obesity-stratified analyses. Most available studies are cross-sectional and therefore provide limited information about whether circulating EV-miRNAs change with tumor progression, treatment, or recurrence. Longitudinal sampling before treatment, during therapy, at response assessment, after treatment, and at recurrence, when feasible, would allow candidate miRNAs to be evaluated in relation to changes in disease status rather than as single time-point associations.

## 10. Limitations

This review should be interpreted considering several limitations. It was conceived as a narrative and integrative review rather than a systematic review or meta-analysis, and therefore does not provide pooled diagnostic, prognostic, or therapeutic effect estimates. In addition, the available literature is highly heterogeneous in sample type, EV isolation strategy, miRNA detection platform, normalization method, obesity definition, metabolic phenotyping, tumor subtype, and clinical endpoints. Many studies also do not clearly distinguish total circulating miRNAs from EV-associated miRNAs or fully control for co-isolated lipoproteins, Argonaute-bound miRNAs, platelet-derived material, hemolysis, and cell death-derived RNA species [149]. Moreover, much of the evidence linking obesity, EV-miRNAs, and breast cancer remains preclinical, associative, or based on small cohorts, with limited functional validation of individual miRNAs as causal mediators. Finally, incomplete reporting of body composition, insulin resistance, menopausal status, diabetes, medication exposure, inflammation, and treatment history limits the interpretation of circulating miRNAs as tumor-derived biomarkers versus mixed host–tumor signals.

## 11. Conclusions

Circulating and EV-associated miRNAs provide a useful framework for understanding obesity-associated breast cancer as a host–tumor communication process rather than as a tumor-only phenomenon. Current evidence is best interpreted along human relevance, with source attribution on one side and mechanistic causality on the other—the two axes used throughout this review—with clinical validation still limited in this setting.

However, their clinical and biological interpretation requires caution because circulating miRNA signals can originate from tumor, adipose, immune, endothelial, platelet, stromal, or mixed sources and can be modified by obesity, metabolic disease, menopausal status, treatment exposure, and pre-analytical variation. Thus, future studies should combine standardized EV-miRNA workflows, metabolic phenotyping, longitudinal sampling, tumor subtype annotation, and functional validation.

In this context, circulating miRNAs should be viewed not only as candidate biomarkers, but also as indicators of how the obese host environment may communicate with breast tumors. This operational framework may help identify clinically meaningful miRNA signatures and therapeutic opportunities aimed at interrupting obesity-driven adipose-tumor signaling without overinterpreting association-only findings as causal mechanisms.

## Figures and Tables

**Table 2:** Minimum biomarker reporting checklist for circulating and EV-associated miRNAs in obesity-associated breast cancer.

Domain	Minimum Reporting Item	Why it Matters in Obesity-Associated Breast Cancer	Key References
Biomarker performance	AUC, sensitivity, specificity, cut-off, 95% CI, and intended use: diagnosis, prognosis, recurrence, or treatment monitoring	Avoids presenting “promising” miRNAs as clinically validated biomarkers without performance metrics	[86,87,115]
Validation design	Discovery cohort, internal validation, external validation, prospective sampling when feasible	Obesity, ethnicity, environment, and clinical background can alter circulating miRNA signatures and reduce transferability	[108,109]
Sample and pre-analytical control	Plasma vs. serum, anticoagulant, fasting status, processing time, centrifugation scheme, platelet depletion or residual platelet assessment, hemolysis check, and freeze–thaw cycles	Platelets, hemolysis, cellular debris, serum/plasma differences, and delayed processing can strongly bias circulating miRNA levels and may mimic tumor- or obesity-associated signals	[116,117]
Analytical workflow	RNA isolation method, exogenous spike-ins, validated endogenous controls, global mean normalization when appropriate, platform used: RT-qPCR, ddPCR, microarray, or small RNA-seq	Normalization and platform choice influence reproducibility; U6/RNU6B should not be assumed stable in extracellular samples unless validated in the same matrix and cohort	[118,119,120]
EV-specific reporting	EV isolation method, EV markers, contaminant markers, particle size/concentration, and distinction between total circulating miRNA and EV-miRNA	Necessary to avoid confusing EV-associated miRNAs with AGO-bound, lipoprotein-associated, platelet-derived, or cell death-derived RNA	[85,121,122]
Obesity/metabolic annotation	BMI plus WC/WHR when available, visceral/subcutaneous adiposity, insulin resistance or diabetes, menopausal status, leptin/adiponectin, CRP, IL-6, TNF-alpha, and relevant medication exposure	Helps distinguish tumor-derived signals from adipose dysfunction, systemic inflammation, endocrine status, and metabolic stress	[83,113,114]
Biological interpretation	State whether the miRNA signal is tumor-derived, adipose-derived, immune/inflammatory, platelet/endothelial, treatment-related, or mixed/uncertain	Prevents overinterpretation of non-specific miRNAs such as miR-21 or miR-155 as tumor-only biomarkers	[123,124,125]

**Table 3:** Human and mechanistic studies of circulating and extracellular vesicle-associated cargoes in breast cancer, annotated by cohort, sample type, obesity or metabolic stratification, and level of validation. The first six entries are biomarker-performance studies; the last three, labelled as mechanistic, are functional EV studies included for comparison and are not diagnostic accuracy studies.

Study (Ref.)	Population/Cohort	Sample and Fraction	Breast Cancer Subtype	Obesity or Metabolic Stratification	Platform	Endpoint and Reported Performance	Independent Validation
Barone et al. [83]	Breast cancer patients stratified by BMI: screening cohort 9 normal weight vs. 18 overweight/obese (pooled, RNA-seq); validation cohort 6 vs. 12 (qRT-PCR).	Serum EVs (ExoQuick); TEM, NTA (94–130 nm), Tsg101/CD9/CD63/CD81/Alix positive, calnexin negative	Mostly ER-positive invasive ductal carcinoma	Yes (BMI-based)	RT-qPCR	EV let-7a-5p lower in overweight/obese patients (*p* = 0.02); inverse correlation with BMI (r = −0.65, *p* = 0.0035); ROC for grade G3 vs. G1/G2 AUC 0.88 (*p* = 0.019); Ki67 AUC 0.64 (*p* = 0.39). No diagnostic accuracy metrics for cancer detection.	Validated in a second cohort of the same study; no broad external validation. The survival association derives from tumor-level expression in public datasets, not from EV measurements.
Shimomura et al. [108]	1280 breast cancer sera (74 training, 1206 test) vs. 2836 non-cancer controls, plus 54 benign breast diseases and 514 other cancers or benign diseases.	Serum, total circulating miRNA	All stages; 256 stage 0 and 483 stage 1	No	Microarray (3D-Gene)	Five-miRNA index (miR-1246, miR-1307-3p, miR-4634, miR-6861-5p, miR-6875-5p): test cohort sensitivity 97.3%, specificity 82.9%, accuracy 89.7%, AUC 0.971; 98.0% detection at stage 0.	Internal test cohort only; 49 of 54 benign breast diseases were also classified as cancer.
Uyisenga et al. [109]	Belgium 143 breast cancers and 136 controls; Rwanda 82 breast cancers and 73 controls (n = 434).	Plasma, total circulating miRNA	Mixed	No	RT-qPCR	Eight-miRNA signature: AUC about 0.80 in Belgium but 0.43 in Rwanda; no signature exceeded AUC 0.6 across sites. A Rwanda-specific signature reached AUC 0.86 (sensitivity 0.92, specificity 0.78) but fell to 0.61 in the Belgian cohort.	Independent cross-site validation; performance not transferable.
Satomi-Tsushita et al. [110]	147 patients with recurrent or metastatic breast cancer pretreated with anthracycline and taxane, receiving eribulin monotherapy (52 with and 95 without new distant metastases).	Serum, total circulating miRNA (2565 miRNAs profiled)	Mixed; 67–75% ER-positive, 85% HER2-negative, 16–21% triple-negative	No	Microarray (3D-Gene) with logistic LASSO and 10-fold cross-validation	Eight-miRNA model predicting new distant metastases after eribulin: AUC 0.79, sensitivity 0.69, specificity 0.82; individual miRNAs AUC 0.62–0.67. miR-8089 and miR-5698 associated with overall survival (multivariable HR 0.61 and 0.76).	None; internal cross-validation only, the authors state the absence of a validation cohort as a limitation.
Wang et al., meta-analysis [115]	16 case–control studies; 1377 breast cancer patients and 716 controls.	Serum and plasma, total circulating miRNA	Mixed	No	Mostly RT-qPCR	Overall: sensitivity 0.93, specificity 0.85, AUC 0.95. Serum: 0.94 and 0.89, AUC 0.97. Plasma (4 studies only): 0.87 and 0.72, AUC 0.73.	Pooled estimates; I2 about 95% for sensitivity and specificity, and Deek’s test indicating publication bias.
Hannafon et al. [122]	16 breast cancer patients vs. 16 women with no history of cancer.	Plasma EVs (ExoQuick, reported as exosomes); electron microscopy, NTA and CD63	Mixed	No	RT-qPCR and small RNA sequencing	Plasma EV miR-1246 (*p* = 0.03) and miR-21 (*p* = 0.04) higher than in controls; AUC 0.69 for each and 0.73 combined (*p* = 0.022); no correlation with stage, grade or tumor size.	No.
Sadegh-Nejadi et al., mechanistic study [104]	5 cancer-free women with obesity (median BMI 34.7) vs. 5 normal-weight donors; MCF-7 recipient cells.	Plasma EVs by differential ultracentrifugation (reported as exosomes); DLS 162 nm, zeta potential −4.07 mV, TEM, CD63	ER-positive model system	Yes (obesity-defined donors)	Functional assays	Increased proliferation (*p* = 0.001), migration (*p* = 0.002), invasion (*p* = 0.038) and MMP-2 (*p*= 0.040) and MMP-9 (*p* = 0.043) activity versus normal-weight EVs; reduced apoptosis under tamoxifen (*p* = 0.013) and lower p53 (*p* = 0.045). miRNA cargo was not measured.	Not applicable; EV-level effects only, miRNA content not analysed.
Xu et al., mechanistic study [22]	96 non-cancer subjects with obesity or overweight vs. 48 normal-weight controls (plasma pooled in groups of 8, mixed sex and predominantly male, so no female-specific analysis was possible); functional work in murine models.	Circulating small EVs, protein cargo (ECM1)	Preclinical models	Yes	Proteomics and functional assays	ECM1 increased in circulating small EVs under obesity; integrin-beta2 controls its loading into the vesicles, not its downstream action. Growth and metastasis effects shown in mice. Supports non-miRNA EV cargo as a mediator; not a biomarker-performance study.	Preclinical.
Llevenes et al., mechanistic study [23]	Murine study: C57BL/6J mice on high-fat diet for 12 weeks vs. low-fat diet controls; syngeneic E0771-GFP cells. No human plasma.	Mouse plasma and adipocyte EVs (reported as exosomes)	Triple-negative	Yes (diet-induced obesity with insulin resistance, in mice)	Functional and transcriptomic analyses	EMT features, increased migration in vitro and increased metastasis in vivo; no diagnostic or human biomarker data.	Preclinical.

Table note: AUC, area under the curve; BMI, body mass index; ECM1, extracellular matrix protein 1; ER, estrogen receptor; EV, extracellular vesicle; MMP, matrix metalloproteinase; RT-qPCR, quantitative reverse transcription polymerase chain reaction. Studies without obesity or metabolic stratification are included because they document the performance reported for circulating miRNA biomarkers. For Satomi-Tsushita et al. the primary source is internally inconsistent: specificity is reported as 0.82 in the abstract, in Table 2 and in the discussion, and as 0.76 in the results text; the value reproduced here is the one that appears in the abstract, the data table and the discussion, and the discrepancy is declared rather than resolved in specific breast cancer settings, and they should be read as indirect evidence for the obesity-associated setting. Performance metrics are reproduced as reported in each source; blank or qualitative entries indicate that the corresponding metric was not reported.

## Data Availability

No new data were created or analyzed in this study. Data sharing is not applicable to this article.

## Supplementary Materials

The following supporting information can be downloaded at https://www.mdpi.com/article/10.3390/cancers18183035/s1. Table S1: Mechanistic map of EV-associated miRNAs and cargoes at the obesity–breast cancer interface, annotated by tumor subtype, obesity phenotype, EV characterization, level of functional manipulation, and main limitations.

## Abbreviations

The following abbreviations are used in this manuscript:

Akt	Protein kinase B
ALIX	ALG-2-interacting protein X
AUC	Area under the curve
BMI	Body mass index
CAA	Cancer-associated adipocyte
CD	Cluster of differentiation
CI	Confidence interval
COX-2	Cyclooxygenase-2
CRP	C-reactive protein
ddPCR	Droplet digital polymerase chain reaction
DGCR8	DiGeorge syndrome critical region 8
DNA	Deoxyribonucleic acid
EGFR	Epidermal growth factor receptor
EMT	Epithelial–mesenchymal transition
ER	Estrogen receptor
EV	Extracellular vesicle
FABP4	Fatty acid-binding protein 4
HER2	Human epidermal growth factor receptor 2
HDL	High-density lipoprotein
HIF-1α	Hypoxia-inducible factor 1-alpha
HOMA-IR	Homeostatic model assessment of insulin resistance
IGF	Insulin-like growth factor
IL	Interleukin
LNA	Locked nucleic acid
lncRNA	Long non-coding RNA
MAPK	Mitogen-activated protein kinase
MCF-7	Michigan Cancer Foundation-7 cell line
MISEV	Minimal Information for Studies of Extracellular Vesicles
MMP	Matrix metalloproteinase
miRNA	MicroRNA
mRNA	Messenger RNA
mTOR	Mechanistic target of rapamycin
NF-κB	Nuclear factor kappa B
NTA	Nanoparticle tracking analysis
OXPHOS	Oxidative phosphorylation
P70S6K	Ribosomal protein S6 kinase beta-1
PLIN1	Perilipin 1
PPARγ	Peroxisome proliferator-activated receptor gamma
PTEN	Phosphatase and tensin homolog
qRT-PCR	Quantitative reverse transcription polymerase chain reaction
REMARK	Reporting Recommendations for Tumor Marker Prognostic Studies
RISC	RNA-induced silencing complex
RNA	Ribonucleic acid
SEC	Size-exclusion chromatography
STARD	Standards for Reporting Diagnostic Accuracy Studies
STAT3	Signal transducer and activator of transcription 3
TEM	Transmission electron microscopy
TNBC	Triple-negative breast cancer
TNF-α	Tumor necrosis factor-alpha
TSG101	Tumor susceptibility gene 101
WC	Waist circumference
WHR	Waist-to-hip ratio
