# Supplementary material for: Circulating microRNAs at the Interface of Obesity and Breast Cancer: From Molecular Machinery, Diagnostic Potential, and Therapeutic Perspectives
Source: Cancers (Basel). 2026 Sep 18;18(18):3035. doi: 10.3390/cancers18183035 (PMC13605481; doi:10.3390/cancers18183035)
Supplement: Supplementary file 1 [file cancers-18-03035-s001.zip › cancers-4549220-supplementary.pdf]

Table S1. Mechanistic map of EV-associated miRNAs and cargoes at the obesity–breast cancer interface, annotated by tumor subtype, obesity phenotype, EV characterization, level of functional manipulation, and main limitations

**miRNA / EV cargo**	**Main axis**	**Phenotype/readout**	**Model/source**	**Evidence type**	**Breast cancer subtype**	**Obesity / metabolic phenotype**	**EV isolation and characterization**	**Functional manipulation and rescue**	**Main limitations**	**References**
let-7a-5p	Obesity-stratified EV-miRNA signal	BMI and tumor grade (the survival association corresponds to tumor-level let-7a in public datasets)	Serum EVs from breast cancer patients	Clinical association (iv/a)	Not restricted (mixed series)	BMI-stratified patients (normal weight vs overweight/obese)	Serum EVs (ExoQuick); TEM, NTA 94-130 nm, Tsg101/CD9/CD63/CD81/Alix positive, calnexin negative	None; no uptake, perturbation or rescue experiments	Association only; cellular origin of the EV signal not established; the let-7a difference was confirmed in a second cohort of the same study, but there is no large external validation. For miR-122 and miR-126 the differences found in the screening cohort were not significant in that second cohort	[83]
miR-155-5p / miR-10a-3p / miR-30a-3p	Akt/mTOR/P70S6K; OXPHOS	Proliferation, mitochondrial activity	Breast adipose EVs from women with obesity	EV-level function plus miRNA-level gain and loss of function (ii/c-d)	ER-positive breast cancer cell models	Human donors with and without obesity (reduction mammoplasty tissue)	EVs from breast adipose tissue explant-conditioned medium by differential ultracentrifugation; TEM, NTA (50-300 nm) and proteomics showing CD9, CD63, CD81 and HSPA8	EV-level function shown; mimics of miR-155-5p, miR-10a-3p and miR-30a-3p reproduced part of the phenotype in recipient cells (miR-155-5p: proliferation and respiration; miR-10a-3p: respiration; miR-30a-3p: proliferation). The vesicles were also pre-incubated with LNA inhibitors against the three miRNAs, which reduced basal respiration, ATP production and maximal respiratory capacity, and reduced proliferation in the	The inhibitors were applied as a mix of the three miRNAs, so the combined loss of function supports a contribution of the group to the respiratory phenotype without resolving the individual contribution of each cargo; P70S6K silencing supports the pathway, not the individual miRNA	[37]

								representative experiment; across seven independent EV preparations the reduction in proliferation did not reach significance		
Adipocyte EV–HIF-1α axis	HIF-1α; mitochondrial metabolism	Growth, invasion, EMT, metabolic adaptation	Adipocyte EVs; overweight/obese patient EVs	EV-level function (ii–iii/b)	ER-positive and triple-negative cell models	Adipocyte differentiation models and overweight/obese patient EVs	EVs from adipocyte-conditioned medium isolated by differential ultracentrifugation and serum EVs isolated with ExoQuick; characterized by TEM, NTA and immunoblotting	EV-level function; pathway modulation shown, no individual miRNA rescue	Mixed sources; obesity phenotyping limited to BMI-based categories	[87,88]
Obesity-associated plasma EVs (reported as exosomes)	MMP activity; endocrine response	Invasion, tamoxifen resistance	Plasma EVs (reported as exosomes) from women with obesity; MCF-7 cells	EVs from cancer-free human donors with obesity, tested on tumor cells; EV-level function (iii/b)	ER-positive (MCF-7)	5 cancer-free women with obesity (median BMI 34.7) vs 5 normal weight	Plasma EVs by differential ultracentrifugation; DLS 162 nm, zeta potential -4.07 mV, TEM, CD63	EV-level function including tamoxifen resistance; miRNA cargo not measured in this study	5 versus 5 cancer-free donors; no metabolic phenotyping beyond BMI; single cell line; no miRNA data	[89]
miR-221/222	ER/p27/PTEN-related resistance pathways	Tamoxifen resistance transfer	Small EVs (reported as exosomes) from resistant breast cancer cells	miRNA-level function, tumor-derived (ii/c)	ER-positive, tamoxifen-resistant and sensitive lines	Not stratified by obesity	EVs from cell-conditioned medium (reported as exosomes)	miRNA-level transfer and phenotype induction demonstrated in [83]; [90] is a separate clinical study of circulating plasma miR-221/222 and does not test	Not obesity-specific; tumor-derived transfer only, no host metabolic context	[98,99]

								EV-mediated transfer		
miR-27a	PPARγ-related insulin resistance	Metabolic dysfunction	Adipocyte-derived small EVs (reported as exosomes) isolated with ExoQuick-TC after pre-filtration; characterized by electron microscopy and CD63 immunoblotting; metabolic models	miRNA-level function, non-breast model (i/c)	Not applicable (skeletal muscle, metabolic model)	Diet-induced obesity and insulin resistance	Adipocyte-derived EVs (reported as exosomes)	miRNA-level gain/loss-of-function in the metabolic model	Indirect for breast cancer; no tumor readout	[90]
miR-34a	Macrophage polarization	Adipose inflammation	Adipocyte-secreted small EVs (reported as exosomes) isolated by ultracentrifugation; characterized by electron microscopy, particle size of 30-100 nm and CD63 immunoblotting; macrophages	miRNA-level function, non-breast model (i/c)	Not applicable (adipose macrophage s)	Diet-induced obesity	Adipocyte-secreted EVs (reported as exosomes)	miRNA-level function on macrophage polarization	Indirect for breast cancer; inflammatory readout only	[89]
Adipose/CAA EV cargo program	Adipose–tumor EV crosstalk	EMT, stromal remodeling, metabolic adaptation	Adipocyte or CAA EV models	Descriptive/ contextual; mixed sources	Mixed models	Variable; often not phenotyped beyond BMI	Heterogeneous isolation methods across sources	Mostly EV-level or descriptive; cargo-specific causality rarely tested	Composite entry drawn from reviews and models; not a single primary dataset	[20,21]

Table note: EV, extracellular vesicle; CAA, cancer-associated adipocyte; EMT, epithelial-mesenchymal transition; ER, estrogen receptor; HIF-1alpha, hypoxia-inducible factor 1-alpha; MMP, matrix metalloproteinase; OXPHOS, oxidative phosphorylation; PPARgamma, peroxisome proliferator-activated receptor gamma; PTEN, phosphatase and tensin homolog. "Evidence type" summarizes the main level of support available in the context discussed and should not be interpreted as definitive clinical validation. The columns report tumor subtype, obesity or metabolic phenotyping, EV isolation and characterization, the level of functional manipulation performed (EV-level function, miRNA enrichment, direct miRNA perturbation, or rescue), and the main limitation of each entry, following the two-axis evidence framework defined in Section 3.1 of the main text. Axis 1 (human relevance and source attribution): (i) non-human or non-breast systems; (ii) human cells or tissue explants; (iii) human biofluids, from breast cancer patients or from cancer-free donors used as an EV source; (iv) human breast cancer cohorts with obesity or metabolic stratification. Axis 2 (mechanistic causality): (a) association only; (b) EV-level function; (c) miRNA-level function; (d) causality with rescue. "Not reported" indicates that the corresponding information was not available in the cited source.
